# Convergent validity and responsiveness of The Standing and Walking Assessment Tool (SWAT) among individuals with non-traumatic spinal cord injury

**DOI:** 10.3389/fneur.2023.1280225

**Published:** 2024-01-23

**Authors:** Mohammad Alavinia, Farnoosh Farahani, Kristin Musselman, Kristina Plourde, Maryam Omidvar, Molly C. Verrier, Saina Aliabadi, B. Catharine Craven

**Affiliations:** ^1^The KITE Research Institute, Toronto Rehabilitation Institute, University Health Network, Toronto, ON, Canada; ^2^Department of Physical Therapy, Faculty of Medicine, University of Toronto, Toronto, ON, Canada; ^3^Institute of Medical Science, Temerty Faculty of Medicine, University of Toronto, Toronto, ON, Canada; ^4^Toronto Rehabilitation Institute, University Health Network, Toronto, ON, Canada; ^5^Rehabilitation Sciences Institute, Faculty of Medicine, University of Toronto, Toronto, ON, Canada; ^6^School of Graduate Studies, University of Toronto, Toronto, ON, Canada; ^7^Department of Medicine, Faculty of Medicine, University of Toronto, Toronto, ON, Canada; ^8^Temerty Faculty of Medicine, University of Toronto, Toronto, ON, Canada

**Keywords:** spinal cord injuries, walking, psychometric properties, outcome assessment, walking speed

## Abstract

**Aim:**

This study aimed to (1) describe the use of the Standing and Walking Assessment Tool (SWAT) among individuals with non-traumatic spinal cord injury or disease (NT-SCI/D); (2) evaluate the convergent validity of SWAT for use among inpatients with NT-SCI/D; (3) describe SWAT responsiveness; and (4) explore the relationship between hours of walking therapy and SWAT change.

**Methods:**

A quality improvement project was conducted at the University Health Network between 2019 and 2022. Participants’ demographics and impairments data, rehabilitation length of stay, and FIM scores were obtained from the National Rehabilitation Reporting System. The walking measure data were collected by therapists as part of routine practice. Hours of part- or whole-gait practice were abstracted from medical records. To determine convergent validity, Spearman’s correlation coefficients were calculated between SWAT stages (admission and discharge) and the walking measures. The change in SWAT levels was calculated to determine responsiveness. Spearman’s correlation coefficient was calculated between SWAT change and hours of walking therapy.

**Results:**

Among adult NT-SCI/D participants with potential walking capacity (SWAT≥1B), the majority were classified as American Spinal Injury Association (ASIA) Impairment Scale D (AIS D) at admission. The SWAT category of 1C (*N* = 100, 18%) was the most frequent at admission. The most frequent SWAT stage at discharge was 3C among participants with NT-SCI/D, with positive conversions in SWAT stages from admission to discharge (*N* = 276, 33%). The mean change in SWAT score was 3 for participants with T-SCI and NT-SCI/D. Moderate correlations between SWAT stages and walking measures were observed. The correlation of hours of gait therapy with the SWAT change (admission to discharge) was 0.44 (*p* < 0001).

**Conclusion:**

The SWAT has sufficient convergent validity and responsiveness for describing standing and walking recovery and communicating/monitoring rehabilitation progress among patients with NT-SCI/D.

## 1 Introduction

Spinal cord injury/D (SCI/D) refers to damage or trauma (motor vehicle accident, fall, and gunshot wound) to the spinal cord resulting in combined or isolated loss or alteration of motor, sensory, and autonomic function at or below the level of cord injury. Non-traumatic spinal cord injury or disease (NT-SCI/D) refers to disease, inflammation, or injury of the spinal cord sufficient to produce motor, sensory, bowel, and bladder impairments, in addition to a plausible non-traumatic etiology of injury (1). The extent of impairment and the specific deficits experienced can vary widely, depending on the location of the injury and the severity of the injury/pathology. The tool most commonly used to predict neurological outcomes after traumatic spinal cord injury (T-SCI) is the International Standards for Neurological Classification of Spinal Cord Injury (ISNCSCI), together with the American Spinal Injury Association (ASIA) Impairment Scale (AIS) (2). The ASIA Impairment Scale is a standardized neurological classification system developed by the American Spinal Injury Association (ASIA) for assessing and categorizing the severity of SCI/D. The scale is based on sensory and motor function, comprehensively describing the extent of impairment following an SCI. The scale ranges from AIS A (complete injury, no motor or sensory function preserved below the level of injury) to AIS E (normal neurological function) (3). Following NT-SCI/D, clinicians seek to predict the functional outcome of patients, help patients understand their anticipated recovery, and ensure the appropriate allocation of rehabilitation resources (4). Among patients with incomplete injuries, walking has been reported as a primary therapeutic goal (5).

The demographic characteristics of individuals admitted for tertiary rehabilitation services in Ontario are changing, with a higher proportion of individuals with NT-SCI/D vs. T-SCI (6). These individuals are more likely to be over the age of 60, female, and have a reasonable prognosis for ambulation based on their admission neurological level, AIS, and lower extremity motor score (LEMS). Many predict that by 2032, individuals aged over 60 will account for 46% of all new injuries, resulting in increases in care costs and rest-of-life costs of 54 and 37%, respectively (7). Therefore, it is critical to consider changes in demographics, etiology, and management of NT-SCI/D when planning for current and future healthcare delivery needs.

Up to 75% of individuals with incomplete SCI/D will experience some gains in walking capacity within the first year following injury (8). Historically, the severity of the injury predicts the amount of recovery in walking following a spinal cord injury (9). Returning to walking is a realistic goal for those with motor incomplete SCI/D or AIS grades C and D and LEMS greater than 20 (9). When a clinical examination is not feasible, such as when a patient is unresponsive, sedated, or uncooperative due to pain, somatosensory evoked potentials can be used to predict motor recovery and walking outcomes (10). Timed measures of walking, such as the 6-min walk test (6MWT) and 10-meter walk test (10MWT), have been the focus of walking assessment in SCI/D rehabilitation and related research settings (11).

The Standing and Walking Assessment Tool (SWAT) is a standardized and objective staging tool used in Canada to evaluate lower limb function in individuals with SCI/D (12), which allows healthcare professionals to track progress, evaluate treatment outcomes, and make informed decisions regarding the choice of appropriate interventions and therapies for each patient. This staging assessment tool allows a therapist to describe a patient’s stage of walking recovery and then guide the choice of walking measures to characterize and evaluate walking abilities during inpatient rehabilitation. The SWAT is usually performed upon rehabilitation admission and discharge or when a patient’s walking ability improves from one state to another. The SWAT includes the 6MWT and 10MWT as two walking measures. The Rick Hansen SCI/D Registry (RHSCIR) introduced the SWAT as a best practice for walking evaluation during inpatient rehabilitation nationally among patients with T-SCI in 2015 (13).

Although the SWAT was implemented as part of the registry for T-SCI, additional efforts were needed to encourage routine walking evaluation of patients with NT-SCI/D during inpatient rehabilitation for all patients admitted among member sites of the Spinal Cord Injury-Implementation and Evaluation Quality Care Consortium (SCI-IEQCC). This included routine implementation of structure, process, and outcome indicators for individuals admitted for tertiary rehabilitation.

The validity, reliability (14), and responsiveness of SWAT among T-SCI have been confirmed in previous studies (15). Convergent validity is the degree to which different measures of the same construct are correlated with each other and refers to the degree to which a measurement tool or instrument accurately measures the theoretical construct or trait it is intended to measure (16). On the other hand, responsiveness was defined as the capacity of the measurement tool of interest to detect changes in a health domain over time (17), specifically changes in standing and walking capacity among inpatients with motor incomplete spinal cord injury.

Despite the higher incidence of NT-SCI/D in Ontario, no studies have described the functional capacity of individuals with NT-SCI/D using SWAT. Given the differences in age, etiology of injury, impairment, and treatment goals among individuals with traumatic and non-traumatic SCI/D and the routine use of SWAT during inpatient rehabilitation in Canada, we sought to: (1) describe the neurological impairments and changes in SWAT staging of patients with NT-SCI/D and T-SCI/D between admission and discharge; (2) evaluate the convergent validity; (3) determine the responsiveness of the SWAT among inpatients with NT-SCI/D; and (4) explore the association between changes in SWAT and hours of evidence-based physiotherapist-delivered whole or partial practice of gait. We hypothesized that the SWAT has sufficient convergent validity and responsiveness for describing standing and walking recovery among patients with NT-SCI/D.

## 2 Materials and methods

### 2.1 Project scope

The data to support the stated objectives were obtained as part of the Spinal Cord Injury Implementation and Evaluation Quality Care Consortium (SCI-IEQCC), an ongoing quality improvement project at the University Health Network (UHN) between January 2019 and December 2022. Descriptions of the procedures for selecting walking as a priority domain for implementation within the SCI-IEQCC (18), and the Spinal Cord Injury Rehabilitation Care High-Performance Indicators (SCI-HIGH) project methods (19), the development of the SCI-HIGH walking indicators (20), the process of concurrent implementation of best practices, and the collection of structure, process, and outcome indicators within the SCI-IEQCC (21) are provided in the referenced manuscripts. Although SWAT was implemented as part of the Rick Hansen Spinal Cord Injury Registry for T-SCI, additional efforts were needed to encourage routine walking evaluation of inpatients with NT-SCI/D using SWAT among member sites of the SCI-IEQCC. This included routine implementation of the walking structure, process, and outcome indicators for all individuals admitted for tertiary rehabilitation. The SCI-IEQCC provided a decision tree depicting when the focus of therapy should be on walking vs. advancing wheelchair skills as the primary goal for functional mobility (22). During the process of routine implementation of the walking indicators, including SWAT as a process indicator, we identified the need to evaluate if the SWAT validity and responsiveness are similar among individuals with NT-SCI/D to those observed among patients with T-SCI (6). As SWAT has been implemented at UHN since 2015 and remains a sustained practice, we chose to conduct this evaluation using UHN data. A Research Ethics Board waiver for the project was obtained (UHN QI # 20-0111).

### 2.2 National rehabilitation reporting system

All adults with NT-SCI/D and T-SCI over 18 years of age admitted for inpatient tertiary SCI rehabilitation at UHN’s Lyndhurst Center were eligible for participation. Participants with Guillain–Barré syndrome (*n* = 1) and multiple sclerosis (*n* = 5) were excluded from the analysis as they did not have a primary cord impairment. Participants were assigned a unique Consortium ID, which was used to de-identify their clinical program SWAT data. This unique ID allowed us to link clinical data with data from the National Rehabilitation Reporting System (NRS) (23) within the central SCI-IEQCC data repository housed at UHN for analysis.

Demographic and impairment data (*n* = 842) for adult inpatients with SCI of traumatic and non-traumatic etiology at the UHN were obtained from the NRS. The NRS collects data from participating adult inpatient rehabilitation facilities and programs across Canada. The following variables were obtained from the local NRS data set: participant’s age, sex, neurological impairment at admission (paraplegia vs. tetraplegia, incomplete vs. complete), rehabilitation client grouping, AIS, admission date, discharge date, length of stay (LOS), and Functional Independence Measure (FIM) scores.

The FIM is an 18-item ordinal scale, scored from 0 to 7, which measures the burden of care and changes in performance throughout a comprehensive inpatient rehabilitation and measures independence in self-care, including sphincter control, transfers, walking, communication, and social cognition (24). This instrument has been used to assess disability among individuals with T-SCI (25). LOS was calculated by subtracting the discharge from the admission date and then subtracting the days the patient was out of the rehabilitation center for any reason (medical assessment and/or emergency room visit). Each participant’s FIM scores at admission and discharge were abstracted. The FIM change and FIM efficiency were calculated. The FIM efficiency score was calculated by subtracting the admission FIM score from the discharge FIM score divided by LOS.

### 2.3 SWAT data collection and analysis

**Figure 1:**
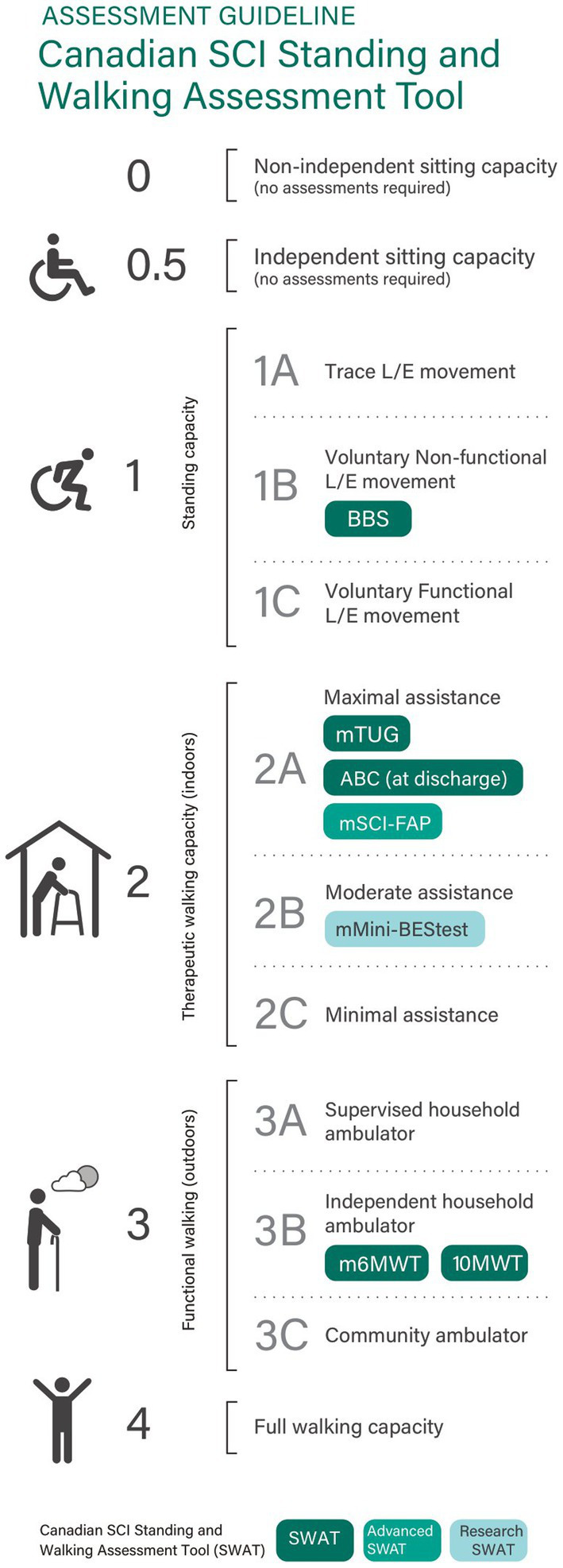
Canadian SCI standing and walking assessment tool (15).

SWAT data were collected for all individuals with T-SCI and NT-SCI/D by therapists as part of routine care within 1 week following inpatient admission and within 1 week before or after discharge. The SWAT stages at admission and discharge were recorded from stages 0 to 4 (Figure 1) (15). The SWAT alphanumeric stage at admission and discharge was recorded and later translated during the analysis into a score from 0 to 11 (creating 12 scores). Thus, SWAT Stage 0 corresponds to a score of 0, and SWAT Stage 4 corresponds to a score of 11 (Table 1). The change in the SWAT stage from admission to discharge was calculated for each participant. Therefore, each unit increase or decrease in SWAT score from admission to discharge was considered a one-category change in SWAT (Figure 1) (15).

**Table 1:** Conversion of the SWAT alphanumeric stage to a SWAT score from 0 to 11.

SWAT stage	0	0.5	1A	1B	1C	2A	2B	2C	3A	3B	3C	4
SWAT score	0	1	2	3	4	5	6	7	8	9	10	11

In T-SCI patients, Musselman et al. reported that individuals with AIS A or B improved by a median of 1 stage (range: 0–11), while those with AIS C and D improved by a median of 3 stages. The responsiveness of SWAT correlated strongly with the Berg Balance Scores and the lower extremity motor scores (*ρ* = 0.778 and 0.836) and moderately with the mTUG (s), 10MWT, and 6MWT scores (15). The 10MWT, which measures walking speed over 10 m at both preferred and maximum speeds, was reported in meters per second (26).

The timed up and go (TUG) is a frequently used outcome measure that evaluates a patient’s ambulatory and transfer skills to determine activity limitations. It has been established that the test’s main objective is to provide an evaluation of overall mobility in patients with a range of disabilities over the lifespan (27). The modified timed up and go (mTUG) test is a variation of the TUG test that accounts for the level of physical assistance required by an individual to complete the test (28). The mTUG, a general measure of mobility, consists of transferring between sitting and standing, walking a short distance, and turning. The time taken to complete the task and the required level of assistance per the procedure specified in the RHSCIR Standing and Walking Toolkit are reported in seconds (13). As we had missing data regarding the assistive device used on the mTUG, only the time in seconds was reported. The mTUG was collected once the patient met the specified threshold for mTUG collection and again at discharge, where appropriate.

### 2.4 Hours of intervention

The ability to move forward over the ground using voluntary lower limb movement while controlling one’s balance in an upright posture (with or without assistance from others or aids) was characterized as walking. The whole or part practice of gait was defined as the below interventions:

- Sit-to-stand training and standing pivot transfers,
- Partial-gait activities, including standing weight-shifting, forced-use exercises, single-leg stance, and stepping,
- Standing balance activities, such as standing in parallel bars without support, perturbations, and walking on soft surfaces,
- Walking activities, including indoor over-ground ambulation, treadmill training with or without body weight support, and outdoor ambulation
- Hydrotherapy activities related to walking goals, gait initiation in water, and walking in waist-height water.
  The time for any interventions mentioned above completed with physiotherapists or physiotherapist assistants was recorded for each patient.

### 2.5 Statistical analysis

A thorough visual examination of raw data and graphical representations were performed for the quality control of the extracted data (*n* = 842). The walking domain analyses were performed for participants with standing (1B standing with assistance) or walking (2A or above) capacity [i.e., SWAT stage 1B and higher (*n* = 442)], and a spinal cord impairment of non-traumatic (*n* = 329) or traumatic etiology (*n* = 113). Participants with a SWAT admission score of 0, 0.5, and 1A (*n* = 391) were excluded from the responsiveness analysis as no change in the SWAT stage was anticipated. All participants with recorded SWAT stages at admission or discharge and etiology for the SCI/D (*n* = 833) were used to calculate the change in SWAT scores. Therefore, if a participant’s stage was 2B upon admission and 3B at discharge, this indicated a three-stage progression. Any negative change from admission to discharge indicates a worsening of the SWAT stage, while a positive number is associated with increments in the SWAT stage.

The appropriate parametric statistical test and independent sample t-tests were used to test the difference between continuous and normally distributed variables, such as age and FIM efficiency score, of the participants with T-SCI and NT-SCI/D. Non-parametric statistics, such as the Spearman correlation coefficient and the chi-square test of independence, were used for hypothesis testing of the severity of injury and level of injury among T-SCI and NT-SCI, as well as SWAT stage at admission and discharge and their walking performance (10MWT PS, 10MWT MS, and mTUG), and compared these two groups. As hypotheses testing for validity assessments should contain an indication of the predicted direction and magnitude of correlations or differences (17), and based on the results from a previous study (15), we expected to detect a positive, moderate correlation between SWAT stage and clinical walking measures (17).

The correlation coefficient measures the strength and direction of the relationship between two variables, with values ranging from −1 to 1. The significant value of ps and bigger correlation coefficients suggest a reliable relationship between the variables. To evaluate responsiveness, the conversion of SWAT from admission to discharge was calculated by the standardized mean difference (Cohen’s *d*). All statistical analyses were performed using R version 4.3.0 (R Foundation for Statistical Computing, Vienna, Austria), considering an alpha error of 0.05. The “effsize Package” in R was used to calculate Cohen’s d and 95% confidence interval.

## 3 Results

### 3.1 Population

We identified a total of 842 participants with SCI/D, including 559 (66%) with NT-SCI/D and 274 (33%) with T-SCI. Nine participants had a missing etiology of injury and were excluded from the analysis; hence, a total of 833 participants were included in the analysis.

### 3.2 Demographic and injury characteristics

The demographic and impairment characteristics of the participants categorized by the etiology of injury are shown in Table 2. Participants with NT-SCI/D were significantly older (*p* < 0.001), with a significantly higher proportion of female participants (*p* < 0.001) compared to those with T-SCI. Data regarding the level of injury were available for 801 participants; among them, 423 (53%) were paraplegic, and 378 (47%) were categorized as tetraplegia. The majority of participants with T-SCI and NT-SCI/D were categorized as AIS D at admission (*n* = 176).

**Table 2:** Characteristics of 833 individuals with SCI/D categorized based on the mechanism of injury.

Variable	Total	Traumatic	Non-traumatic	*p*-value
	(*N* = 833)	(*N* = 274)	(*N* = 559)	
Age – mean ± SD	58.48 ± 16.88	53.93 ± 18.73	60.64 ± 15.43	<0.001
Female – *N* (%)	296 (35.2)	64 (23.4)	230 (41.1)	<0.001
LOS – mean ± SD	63.11 ± 34.80	71.81 ± 36.05	59.61 ± 32.98	<0.001
Severity incomplete	743 (89.2)	219 (79.9)	524 (93.7)	<0.001
FIM efficiency score – Mean ± SD	0.66 ± 1.22	0.60 ± 0.70	0.75 ± 0.83	0.008
^*^Injury severity – N (%)				
AIS A – Complete	71 (11.8)	53 (19.4)	18 (5.6)	<0.001
AIS B – Incomplete	38 (6)	24 (8.8)	14 (4.3)	
AIS C – Incomplete	102 (17)	48 (17.6)	52 (16.1)	
AIS D – Incomplete	389 (64.7)	148 (54.2)	238 (73.7)	
AIS E – Normal	1 (0.2)	0 (0)	1 (0.3)	
Level of injury – N (%)				
Paraplegia	423 (53)	101 (37)	322 (52)	0.00009
Tetraplegia	378(47)	169 (63)	299 (48)	

^*^For 236 participants with non-traumatic spinal cord injury or disease (NT-SCI/D) and 1 participant with traumatic spinal cord injury, AIS data were not available.

### 3.3 SWAT analysis

**Figure 2:**
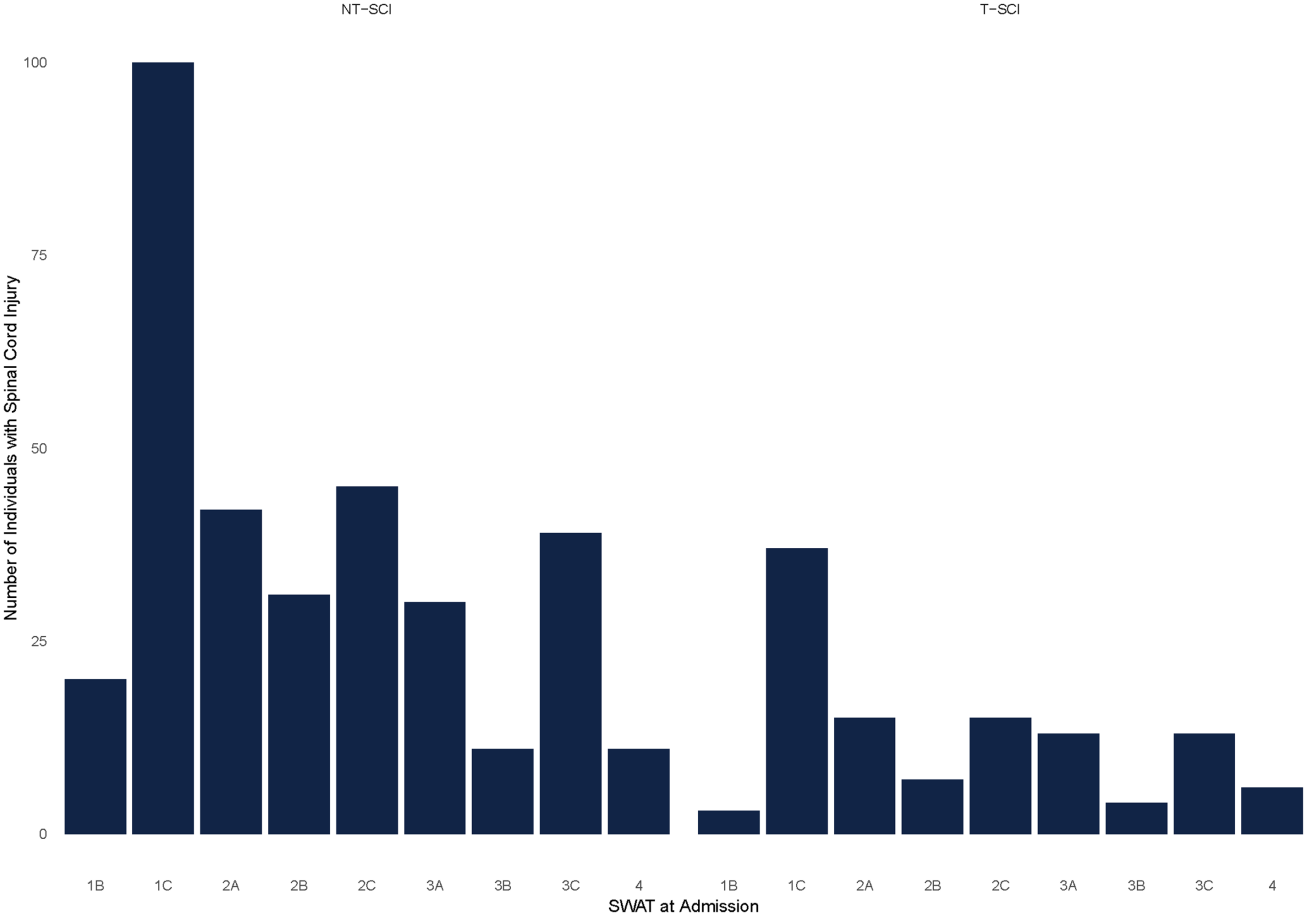
SWAT at admission by the mechanism of injury (trauma vs. non-trauma).

**Figure 3:**
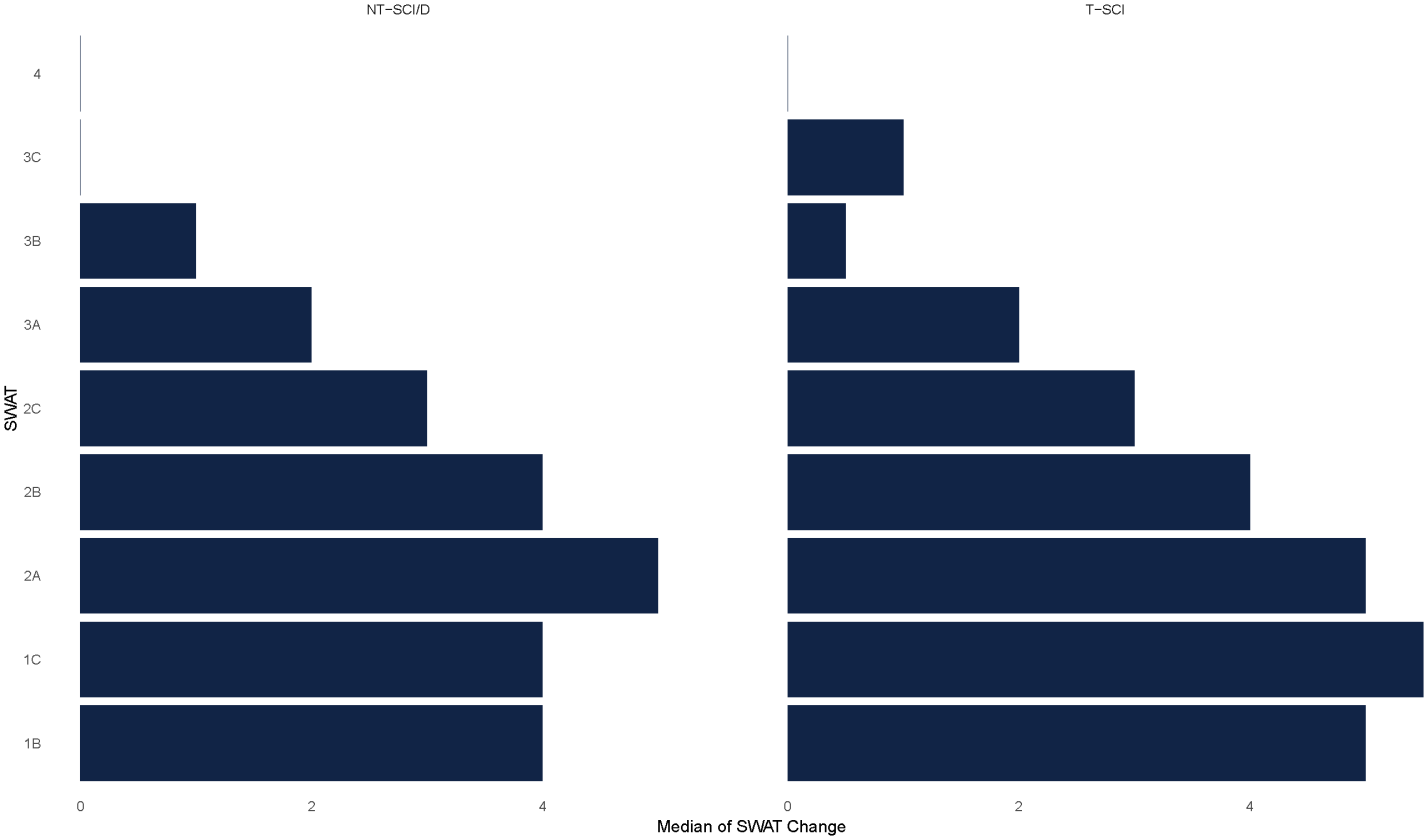
Median of SWAT change from admission to discharge by injury etiology.

Forty-eight percent (*N* = 366) of participants with NT-SCI/D or T-SCI had a SWAT stage of 0, 0.5, and 1A at admission, indicating no standing and/or walking capacity, and were excluded from the subsequent analyses. The remaining participants with NT-SCI/D with SWAT data (*n* = 329) vs. T-SCI (*n* = 113) were included in the analysis of the relationship between the SWAT stage and walking performance (Figure 2). The mean changes in SWAT score from admission to discharge for NT-SCI/D and T-SCI were 2.94 (SD = 2.09) and 3.22 (SD = 2.06), respectively. Cohen’s d effect size was −1.38 (95% CI: −1.54, −1.23) among NT-SCI/D with walking capacity, suggesting a large practical significance and highlighting the magnitude of the observed difference between the SWAT at admission and SWAT at discharge. The negative Cohen’s *d* suggests that the mean of the SWAT at admission is lower than the mean of the SWAT at discharge. The mean change for each SWAT stage was slightly lower in participants with NT-SCI/D vs. T-SCI; however, the differences were not statistically significant (*p* = 0.47). Figure 3 depicts the median SWAT scores categorized by SWAT at admission.

**Figure 4:**
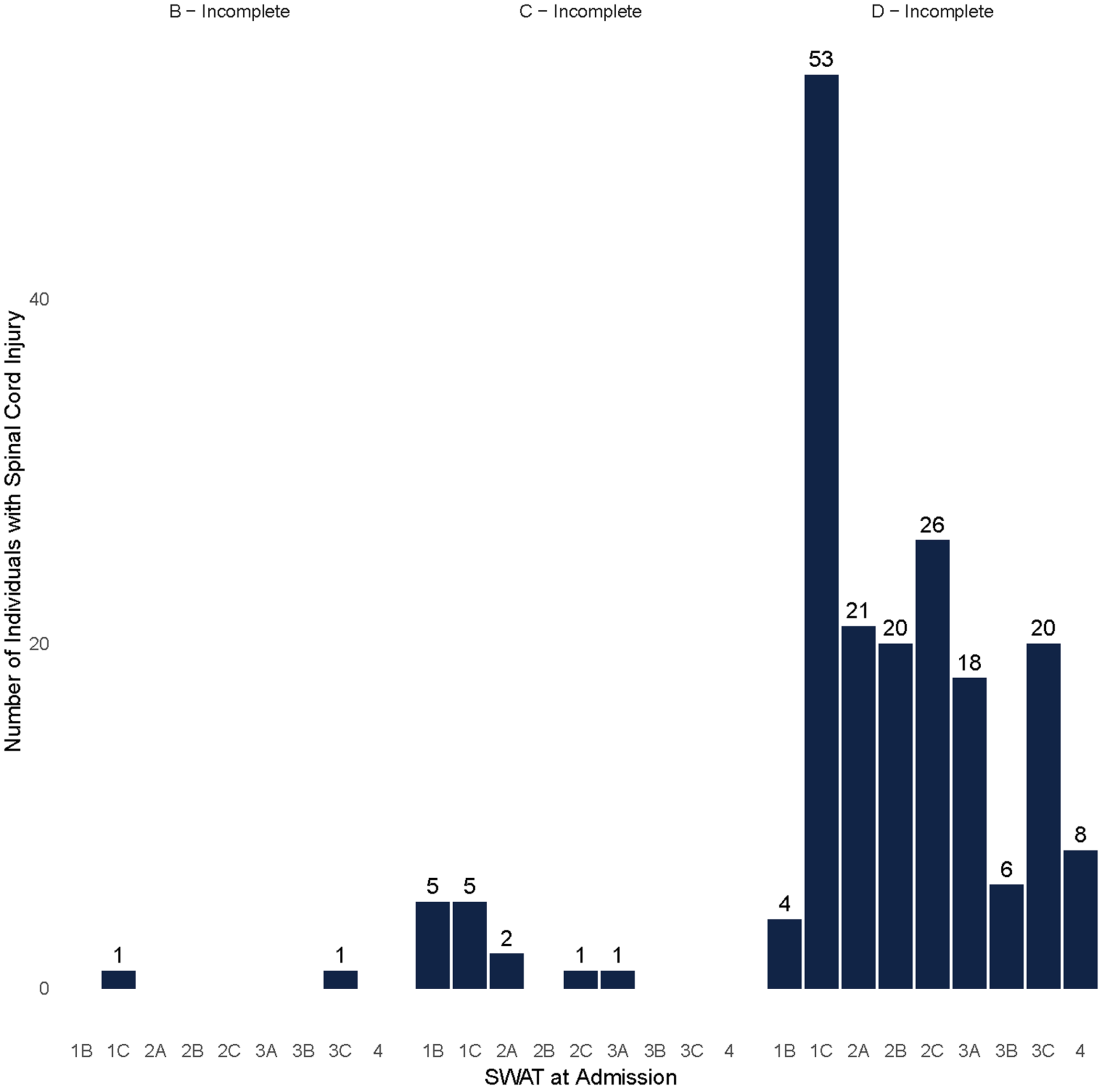
SWAT at admission by AIS among non-traumatic individuals with SCI/D who had the capacity to walk (SWAT*≥*1B).

**Figure 5:**
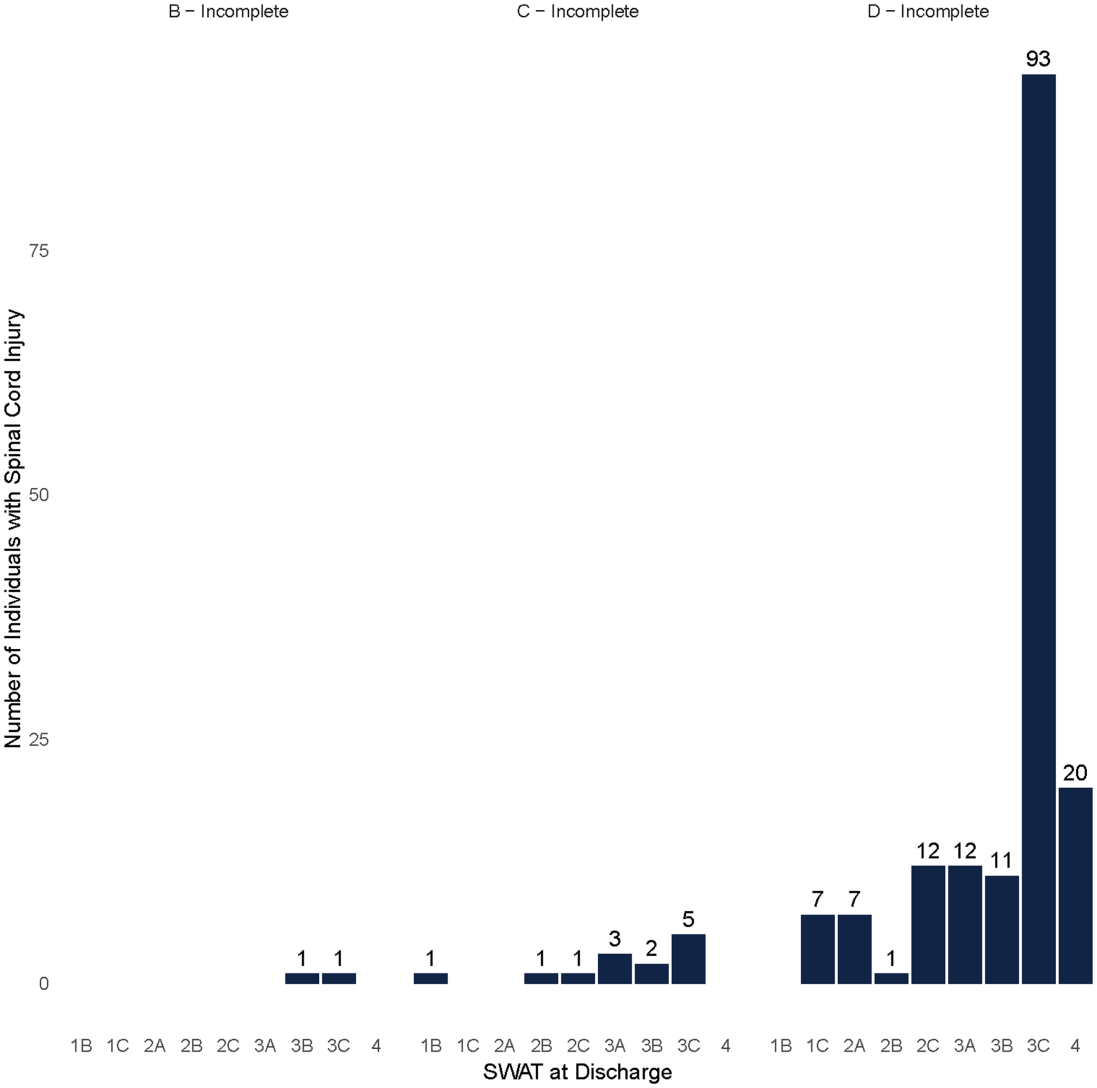
SWAT at discharge by AIS among non-traumatic individuals with SCI/D who had the capacity to walk (SWAT*≥*1B).

Among participants with NT-SCI/D with potential capacity to stand or walk (SWAT 1B and higher), the majority were classified as AIS D at admission (*N* = 176), with the highest percentage belonging to the SWAT category of 1C (*N* = 53, 28% of NT-SCI with potential capacity to stand or walk) (Figure 4). The most frequent SWAT stage at discharge was 3C among participants with NT-SCI/D (*N* = 93, 52% of NT-SCI with potential capacity to stand or walk), with positive conversions in SWAT stages from admission to discharge observed among most participants (*N* = 252, 82%) (Figure 5). A similar frequency of SWAT category 3C at discharge was observed among participants with T-SCI (shown = 48, 46%). A total of 69 participants (both NT-SCI/D and T-SCI) had no observed change in SWAT. One participant had a one-level deterioration in the staging score (SWAT score change = −1) throughout rehabilitation (admission SWAT = 2A). The mean (SD) of the SWAT change among participants with paraplegia and tetraplegia of non-traumatic etiology in terms of walking capacity was 2.91 (2.06) and 3.06 (2.93), respectively.

### 3.4 Correlation between walking measures and SWAT

Table 3 presents the frequency of the SWAT stage at admission and discharge. The distribution of 10 MWT Preferred Speed (10MWT PS), 10 MWT Maximum Speed (10MWT MS), and mTUG scores (all in seconds) show a distribution slightly skewed to the right at admission and discharge for participants with NT-SCI/D. However, these scores for different SWAT stages improved from admission to discharge (Table 4). The mean of 10MWT PS (Meter/Sec) was slightly higher in tetraplegic participants (0.70 ± 0.25) compared to participants with paraplegia (0.62 ± 0.30).

**Table 3:** SWAT at admission and discharge for the total cohort of participants categorized by injury etiology, prior to the exclusion of those without walking potential (less than 1B) – N (%).

SWAT	Admission – N (%)			Discharge – N (%)		
	Total	Traumatic	Non-traumatic	Total	Traumatic	Non-traumatic
	(*N* = 809)	(*N* = 272)	(*N* = 533)	(*N* = 763)	(*N* = 252)	(*N* = 507)
0*	202 (25)	100 (37)	100 (19)	67 (9)	38 (15)	28 (5)
0.5*	114 (14)	43 (16)	71 (13)	57 (8)	28 (11)	29 (6)
1A*	50 (6)	16 (6)	33 (6)	59 (8)	21 (8)	38 (7)
1B	23 (3)	3 (1)	20 (4)	18 (2)	7 (3)	11 (2)
1C	137 (17)	37 (14)	100 (19)	37 (5)	8 (3)	29 (6)
2A	57 (7)	15 (5)	42 (8)	26 (3)	8 (3)	18 (4)
2B	38 (5)	7 (3)	31 (6)	15 (2)	4 (2)	11 (2)
2C	60 (8)	15 (5)	45 (8)	57 (8)	14 (6)	42 (8)
3A	44 (5)	13 (5)	30 (6)	48 (6)	11 (4)	35 (7)
3B	15 (2)	4 (1)	11 (2)	40 (5)	15 (6)	25 (5)
3C	52 (6)	13 (5)	39 (7)	276 (36)	74 (29)	202 (40)
4	17 (2)	6 (2)	11 (2)	63 (8)	24 (10)	39 (8)

**Table 4:** Mean and SD categorized by SWAT stage at admission and discharge for 10MWT PS, 10 MWT MS, and mTUG among NTSCI/D Participants.

SWAT stage	10MWT PS (Sec)		10MWT MS (Sec)		mTUG (Sec)	
	Admission	Discharge	Admission	Discharge	Admission	Discharge
1B	31.95 ± 24.11	26.37 ± 20.73	28.95 ± 21.28	23.40 ± 18.76	30.92 ± 26.95	18.25 ± 8.38
1C	32.62 ± 23.08	23.48 ± 12.49	21.18 ± 13.25	17.06 ± 7.59	31.35 ± 24.05	25.51 ± 16.14
2A	21.07 ± 9.31	17.65 ± 10.55	17.04 ± 10.25	13.01 ± 7.63	21.93 ± 10.44	19.81 ± 18.24
2B	17.10 ± 3.40	17.39 ± 6.40	12.08 ± 2.22	13.13 ± 5.31	21.52 ± 8.06	18.82 ± 13.05
2C	17.85 ± 7.04	19.19 ± 10.07	12.77 ± 3.96	13.57 ± 6.61	25.70 ± 14.61	18.72 ± 10.90
3A	20.62 ± 15.38	26.44 ± 40.83	15.16 ± 11.58	13.51 ± 10.20	20.72 ± 18.74	13.06 ± 13.85
3B	17.30 ± 5.99	12.49 ± 4.59	12.53 ± 3.87	9.22 ± 3.01	18.41 ± 12.77	9.07 ± 5.28
3C	12.95 ± 5.04	10.60 ± 3.12	9.91 ± 4.06	7.87 ± 2.33	13.15 ± 5.85	9.65 ± 5.45
4	10.44 ± 2.10	8.69 ± 1.64	7.18 ± 1.76	6.13 ± 0.88	10.74 ± 3.23	4.59 ± 5.75

The findings in Table 5 display the Spearman’s Rho correlation coefficients and corresponding value of ps for various measures, including the 10MWT PS, 10MWT MS, mTUG time, total FIM at admission, FIM efficiency score, LOS, and hours of walking service intervention, and the SWAT scores at admission and discharge among participants with NT-SCI/D. The SWAT admission scores positively correlated with the 10MWT PS and the 10MWT MS and negatively correlated with the mTUG test. These correlation coefficients suggest that higher SWAT admission scores are associated with better performance (lower time or higher speed) on these measures. The same directionality for the correlation, with a slightly weaker correlation, was observed at discharge. The FIM efficiency scores also revealed a positive correlation with the SWAT admission scores, suggesting that higher efficiency in completing activities of daily living is associated with higher SWAT admission scores (Spearman’s Rho = 0.43, *p* < 0.0000). The mean total hours of gait practice with physiotherapists or physiotherapist assistants were 10.44 (SD ± 8.05) in participants with NT-SCI/D, with the highest hours devoted to participants with SWAT stages 1C and 2A (13.04 and 13.92 h, respectively). The correlation of hours of gait intervention with the SWAT change from admission to discharge (not shown) was 0.44 (*p* < 0.0001). The hours of gait practice showed a negative correlation with the SWAT admission scores, suggesting that higher hours of gait intervention from physiotherapists are devoted to the participants with potential walking capacity and lower SWAT stages (1B, 1C, and 2A) (Spearman’s Rho = −0.44, *p* < 0.00001).

**Table 5:** Correlation coefficients (Spearman’s Rho) and corresponding value of ps for various measures and SWAT scores at admission and discharge among individuals with non-traumatic SCI.

		Spearman’s Rho	*p*-value
SWAT admission	Admission		
	10MWT PS	0.57	<0.0001
	10MWT MS	0.61	<0.0001
	mTUG	−0.52	<0.0001
	Total FIM – Admission	0.65	<0.0001
	FIM efficiency score	0.59	<0.0001
	LOS	−0.63	<0.0001
	Hours of walking service intervention	−0.44	<0.0001
SWAT discharge	Discharge		
	10MWT PS	0.37	<0.0001
	10MWT MS	0.56	<0.0001
	mTUG	−0.43	<0.0001
	Total FIM – discharge	0.77	<0.0001
	FIM efficiency score	0.69	<0.0001
	LOS	−0.6	<0.0001
	Hours of walking service intervention	−0.23	0.004

## 4 Discussion

As hypothesized, the convergent validity and responsiveness of SWAT among participants with NT-SCI/D were moderate and similar to those previously observed in the Canadian T-SCI population (15). Therefore, in addition to the participants with T-SCI, SWAT stages are appropriate to describe walking recovery among individuals with NT-SCI/D. The study results revealed a moderate correlation between SWAT stages and walking measures, including 10MWT PS, 10MWT MS, mTUG, and FIM, among participants with NT-SCI/D. The correlation coefficients between SWAT and 10MWT PS and 10MWT MS were slightly higher than those reported for SWAT stages and walking measures in T-SCI (15). These findings and Cohen’s d statistics support the convergent validity and responsiveness of the SWAT as a measure of standing and walking ability in individuals with NT-SCI/D.

This study’s strength is the substantial and diverse sample of participants with NT-SCI/D. This enables the authors to draw meaningful and generalizable conclusions, enhancing the reliability and validity of the findings. Furthermore, the quality improvement work verifies the relationship between the SWAT stage and walking performance, 10MWT MS (m/s), 10MWT PS (m/s), and mTUG (s).

We acknowledge that LOS and hours of service provision are driven by many variables. For example, among participants whose admission SWAT stage is 1B-2A, there tend to be more global mobility needs, including equipment and housing. Therefore, a longer LOS is required, and more hours of gait practice are provided. Despite these provisos, the assessment of hours of gait practice showed a moderate and positive correlation with SWAT change, indicating a longer duration of intervention during rehabilitation results in a greater change in SWAT staging. The data exploring the relationship between SWAT change and hours of walking intervention reveal that physiotherapists and physiotherapy assistants are appropriately spending more time with participants (spent on transfers, pre-gait activities, and aquatic therapy with the lower functioning participants) with walking potential and lower SWAT stages at admission.

Categorizing individuals with SCI/D using SWAT is a standardized and comprehensive walking assessment (12). The 10MWT is a valid and reliable outcome measure to assess walking speed over a short distance in participants with SCI/D (29). Among the three measurements, mTUG had the lowest correlation with the SWAT scores at admission, likely due to the heterogeneity in trunk control. However, all walking measures showed a moderate correlation with SWAT at admission and discharge. The correlation coefficient for 10MWT MS was 0.61 at admission and 0.56 at discharge, slightly higher than the moderately positive correlation between 10MWT PS and the SWAT scores. The SWAT has the potential to more accurately reflect the capacity for performing activities of daily living (e.g., supervised household ambulator and community ambulator) (12). Similar to the findings of this study, among T-SCI participants, Musselman et al. (15) found that SWAT stages are moderately correlated with mTUG, 10MWT, and 6MWT.

The Spinal Cord Independence Measure III (SCIM) and FIM are two essential assessment tools used in SCI/D rehabilitation to evaluate functional abilities and describe the burden of care among individuals with SCI/D, respectively. The SCIM is a specialized instrument explicitly designed to assess various aspects of functional independence related to activities of daily living in individuals with spinal cord lesions. SCIM takes into account tasks such as self-care, mobility, and respiration. The SCIM and FIM play crucial roles in guiding treatment plans, tracking progress, and determining the level of assistance and support needed by individuals with spinal cord injuries to achieve optimal functional outcomes and improve their overall quality of life. The FIM is a multi-dimensional scale with a motor subscale that includes two locomotor-related items: walking or wheelchair propulsion and stair climbing. FIM is intended to assess the burden of care and functional impairment and is not a pure ambulation measure (30). For assessing patients’ functional independence with SCI/D, SCIM III is likely a better measure (31). However, in this quality improvement project, only FIM total scores were available for analysis. Therefore, finding a moderate correlation between SWAT and FIM at admission and discharge is not surprising.

The study results verify that the etiology of SCI (NT-SCI/D vs. T-SCI/D) is not a predictor of AIS improvement during rehabilitation (32). However, other authors have proposed that patients with T-SCI suffer from more severe neurologic impairments compared to patients with NT-SCI/D (33). Although age is not associated with functional recovery after rehabilitation for SCI/D (34), individuals with NT-SCI/D tend to be older, and their recovery takes longer compared to younger individuals. Future studies developing recovery profiles for patients with NT-SCI/D should adjust for age at injury, cord pathology, and duration of injury. Most participants with a walking capacity based on SWAT stages were in the AIS D group with NT-SCI/D. The distribution of SWAT at admission and discharge was not different between participants with T-SCI and NT-SCI/D who converted to SWAT stage 3C prior to discharge. Considering most participants were at stage 1C at admission, the conversion to stage 3C represents a 6-level improvement in the SWAT stage at discharge. Although the SWAT stages showed a floor effect among individuals with T-SCI (12), this was not evident among NT-SCI/D participants in this quality improvement project, as we removed those at stages 0, 0.5, and 1A.

Some limitations should be considered when interpreting the results of this quality improvement project. We used data from the clinical practice of local healthcare providers in a tertiary rehabilitation center. The project plan and analysis were not developed *a priori*. Second, the subscales of FIM were not available in our admin data set and thus were not included in the data analysis, which would have been a preferable strategy. However, the correlations of SWAT with the FIM total score and FIM efficiency score were measured. Third, the number of missing values for AIS was high among participants with NT-SCI/D patients, reflecting the complexity of ISCNSCI reporting in this group. This might have influenced the distribution of the SWAT stages across AIS groups. However, the assessment of the missing values did not reveal important differences or a systematic bias with the available data, as the missing values were likely random. Fourth, we did not calculate mTUG scores; rather, we reported the mTUG time in seconds. Fifth, information about the lower extremity motor score was not available, making it impossible to describe changes in motor scores with concurrent changes in SWAT stages. Finally, the absence of the 6-min walk test and Berg Balance Scale score data is also a limitation of this study. However, these measures are best used among those with established walking ability and may not be most appropriate during inpatient rehabilitation when the focus of care is on pre-gait or early gait interventions. On the other hand, the Berg Balance Scale does not consider dynamic balance and only assesses postural changes, transfers, static balance, and activities not strictly related to walking (35).

In conclusion, the SWAT has sufficient convergent validity and responsiveness for describing standing and walking recovery among patients with NT-SCI/D. The findings suggest that higher SWAT scores at admission and discharge are associated with better performance on measures of walking ability (10MWT and mTUG) and higher functional independence. These results support the use of the SWAT as a tool for assessing and tracking walking capacity and functional outcomes in participants undergoing inpatient rehabilitation with NT-SCI/D. SWAT staging brings together commonly used measures of walking and balance and may, in the future, provide some guidance regarding the optimal timing and intensity of rehabilitation and be a valuable tool for describing recovery during rehabilitation among clinicians. SWAT addresses the requirement for a uniform method of evaluating the lower extremities appropriate for all individuals with SCI by demonstrating the validity of this categorization system among participants with NT-SCI/D. By implementing the SWAT, clinicians can gather valuable data to monitor changes in walking ability over time, inform minimum service requirements, and contribute to efforts to improve our therapeutic interventions to augment walking outcomes and limit related impairments in individuals with SCI.

## Data availability statement

The data analyzed in this study is subject to the following licenses/restrictions: access to the dataset is restricted to authorized individuals or groups. Users are required to obtain permission or credentials to access and use the dataset. Requests to access these datasets should be directed to BC at cathy.craven@uhn.ca.

## Ethics statement

Ethical approval was not required for the study involving humans in accordance with the local legislation and institutional requirements. Written informed consent to participate in this study was not required from the participants or the participants’ legal guardians/next of kin in accordance with the national legislation and the institutional requirements.

## Author contributions

MA: Data curation, Formal analysis, Methodology, Software, Visualization, Writing – original draft, Writing – review & editing. FF: Project administration, Writing – review & editing. KM: Writing – review & editing. KP: Data curation, Writing – review & editing. MO: Data curation, Writing – review & editing. MV: Writing – review & editing. SA: Formal analysis, Writing – review & editing. BC: Conceptualization, Funding acquisition, Investigation, Methodology, Project administration, Resources, Supervision, Validation, Writing – original draft, Writing – review & editing.
